# Supplementary material for: Rhesus Cytomegalovirus-encoded Fcγ-binding glycoproteins facilitate viral evasion from IgG-mediated humoral immunity
Source: Nat Commun. 2025 Jan 31;16:1200. doi: 10.1038/s41467-025-56419-3 (PMC11782611; doi:10.1038/s41467-025-56419-3)
Supplement: Supplementary file 1 — Supplementary Information [file 41467_2025_56419_MOESM1_ESM.pdf]

Rhesus Cytomegalovirus-encoded Fcγ-binding glycoproteins facilitate viral evasion from IgG-mediated humoral immunity

Claire E. Otero[bc#], Sophia Petkova[a#], Martin Ebermann[a#], Husam Taher[d], Nessy John[d], Katja Hoffmann[a], Angel Davalos[f], Matilda J. Moström[e], Roxanne M Gilbride[d], Courtney R. Papen[d], Aaron Barber-Axthelm[d], Elizabeth A. Scheef[e], Richard Barfield[f], Lesli M. Sprehe[e], Savannah Kendall[e], Tabitha D. Manuel[e], Teresa Beechwood[d], Linh Khanh Nguyen[d], Nathan H. Vande Burgt[d], Cliburn Chan[f], Michael Denton[d], Zachary J. Streblow[d], Daniel N.Streblow[d], Alice F. Tarantal[g], Scott G Hansen[d], Amitinder Kaur[e], Sallie Permar[b], Klaus Früh[d], Hartmut Hengel[a], Daniel Malouli[d*], Philipp Kolb[a*]

[a]Institute of Virology, Medical Center, Faculty of Medicine, University of Freiburg, Freiburg, Germany
[b]Department of Pediatrics, Weill Cornell Medicine, New York, New York, United States of America
[c]Department of Pathology, Duke University, Durham, North Carolina, United States of America
[d]Vaccine and Gene Therapy Institute, Oregon Health and Science University, Beaverton, Oregon, United States of America
[e]Tulane National Primate Research Center, Tulane University, Covington, Louisiana, United States of America
[f]Department of Biostatistics and Bioinformatics, Duke University, Durham, North Carolina, United States of America
[g]Departments of Pediatrics and Cell Biology and Human Anatomy, School of Medicine, and California National Primate Research Center, University of California, Davis, CA, United States of America
[*,#]These authors contributed equally to this work.

Short Title: RhCMV evasion of humoral immunity.

*Co-corresponding authors:
Daniel Malouli
Vaccine and Gene Therapy Institute,
Oregon Health and Science University,
505 NW 185th Ave., Beaverton, OR 97006.
Phone: (503) 418-2738
Fax: (503) 418-2701
E-mail: maloulid@ohsu.edu

Philipp Kolb
Institute of Virology,
Medical Center, Faculty of Medicine,
University of Freiburg,
Hermann-Herder-Str. 11
79104 Freiburg, Germany
Phone: +49 761 203-6587
Fax: +49 761 203-6626
E-mail: philipp.kolb@uniklinik-freiburg.de

Supplementary Tables and Figures

Table S1. Primers and probes for the RT-qPCR/RT-PCR analyses.

**Rh 03.1**	Forward	5’ GGCTGCTGAACGCAAGCT 3’
	Reverse	5’ TGTAGGCAAACAGGAACAGCAA 3’
**Rh05**	Forward	5’ AGTATCGGCCGCTGAATGAG 3’
	Reverse	5’ TCCCGTCCGGAAAATCG 3’
	Probe	6FAMACCCTGCGCCTCAGMGBNFQ
**Rh06**	Forward	5’ TGAAAAAGCGCCATAAAAATTCT 3’
	Reverse	5’ TGGTTGGGAATACAAGTTGAATCC 3’
**Rh150**	Forward	5’ GCAACGCCTTTCAGAACCTT 3’
	Reverse	5’ AGGACGGTGATGTTGTGCAA 3’
**Rh152/151**	Forward	5’ TCGGAACACTGGGCTATATCG 3’
	Reverse	5’ ACAGAAGGGCAAATAGCATGAAG 3’
	Probe	6FAMCTTGGCCTTCCTCCMGBNFQ
**Rh154**	Forward	5’ CTGAACTGGTGGCCTCGAA 3’
	Reverse	5’ AGTTGCGCTAACAGAACAATCATC 3’
**Rh172**	Forward	5’ GGCGGCGATCGCATT 3’
	Reverse	5’ CCACTCTCTGGTTCGCTTCAT 3’
**Rh173**	Forward	5’ ATACTACCGTCAACGCCACTCA 3’
	Reverse	5’ TTCTCTCTGGACGGTATCGAACA 3’
	Probe	6FAMACGACACAGAACAGCMGBNFQ
**Rh174**	Forward	5’ GCTCCGGCTGCCACAA 3’
	Reverse	5’ CAGTGATGCAACGCACTTCCT 3’

Table S2. ***In vivo*** **viral control.**

Viral load was measured via qPCR targeting exon 1 of the IE locus. For each animal in the time to viral control analysis, we indicate the vFcγR status of the inoculation strain(s), the first time point at which a detectable viral load was measured, the time point at which viral load peaked, the copy number observed at the peak time point, the time point at which viral load was no longer detectable after the peak time point, the last time point that a plasma sample was collected, and the estimated day that DNAemia was controlled calculated as the midpoint between the first post-peak time point below the limit-of-detection and the previous time point. If DNAemia was not controlled by the end of the sampling period, the day first below limit-of-detection beyond peak was listed as “N/A” and censoring time point is listed in the final column.

Animal ID	vFcγR	Day first VL above LOD	Day peak VL	VL peak (copies/mL)	Day first below LOD beyond peak	Last day observed	Day viremia controlled or last observed
004-101	Deleted	7	7	3500.8	35	42	31.5
004-102	Deleted	7	7	173411.6	28	42	24.5
004-103	Deleted	7	7	14289.9	35	49	31.5
004-104	Deleted	2	7	116343.3	49	49	45.5
001-101	Intact	1	7	74209.7	N/A	98	98.0
001-102	Intact	1	14	27457.9	97	105	91.0
001-103	Intact	7	7	39040.0	47	86	43.5
001-104	Intact	1	14	67117.4	63	98	59.5
001-105	Intact	1	4	21040.9	63	98	59.5
001-106	Intact	1	14	36751.1	49	98	45.5
001-107	Intact	4	7	137154.6	42	105	38.5
001-108	Intact	1	14	98403.0	56	105	52.5
001-109	Intact	1	7	38366.7	49	98	45.5
001-110	Intact	1	7	17453.0	91	98	87.0
001-111	Intact	4	7	86682.0	83	83	80.0
001-112	Intact	1	7	57974.5	49	98	45.5
002-101	Intact	7	7	133027.0	N/A	65	65.0
002-102	Intact	2	10	10145.0	65	65	61.0
002-103	Intact	7	10	122668.0	N/A	64	64.0

Abbreviations: vFcγR: viral Fc gamma receptor. VL: viral load. LOD: limit-of-detection.

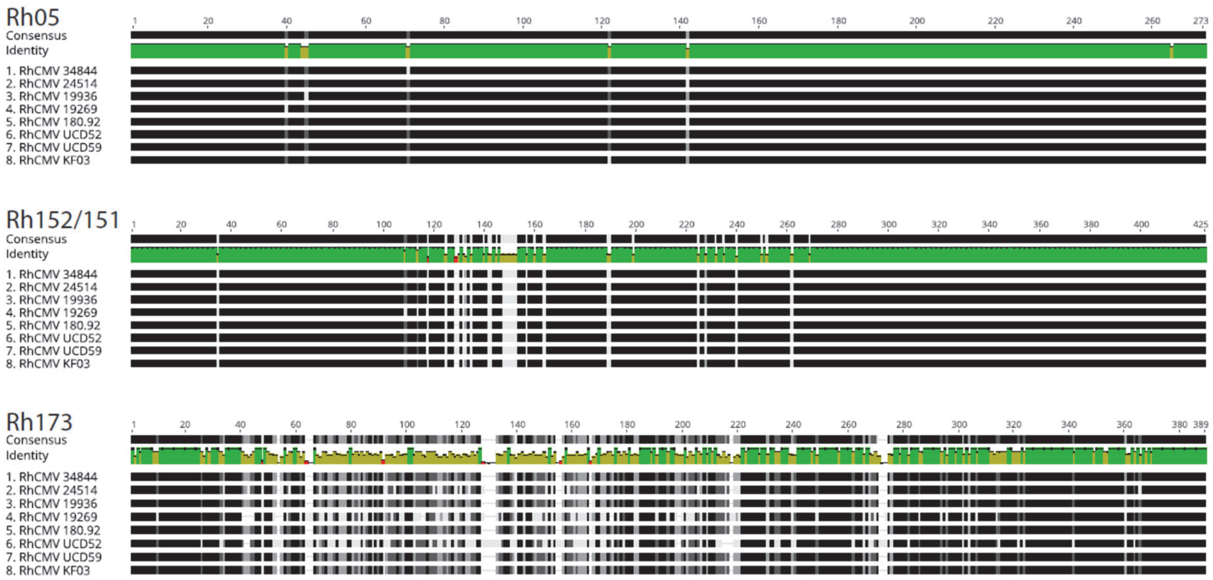

Fig S1. RhCMV vFcγR amino acid sequence alignments.
Amino acid sequence alignments of RhCMV vFcγRs across all published full-length RhCMV genome sequences (refSeq NCBI) from primary isolates and excluding laboratory adapted strain sequences.

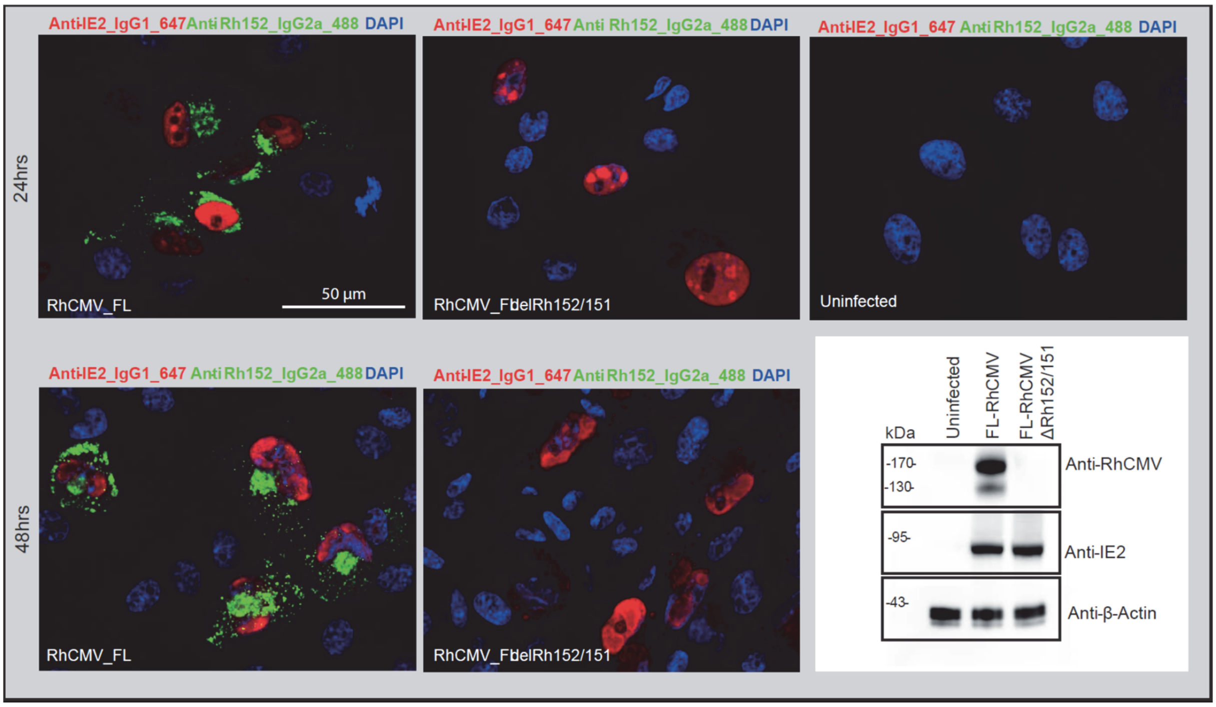

Fig S2. A RhCMV-specific mAb recognizes Rh152/151.
A mAb from a screen of RhCMV specific mouse hybridoma cell lines was tested for RhCMV protein specificity. tRFs were infected with FL-RhCMV or FL-RhCMVΔRh152/151 at MOI 1. IFA was performed at 24 hpi and 48 hpi using anti-IE2 and anti-RhCMV antibodies for detecting infected cells. Uninfected tRFs show absence of fluorescence signals, while at 24 hpi and 48 hpi cells show nuclear IE2 staining. The RhCMV-specific antibody displayed a punctate patterns at both time points. In contrast, FL-RhCMVΔRh152/151-infected cells displayed a loss of RhCMV-specific antibody binding, while preserving IE2 detection. This indicated that the RhCMV-specific antibody recognizes the Rh152/151 protein in RhCMV infected cells. This conclusion was confirmed by immunoblot analysis where the same RhCMV-specific mAb detected a protein band that was absent from FL-RhCMVΔRh152/151-infected cell lysates. IE2 and β-actin were included as infection and loading control, respectively. One of two independent experiments. Source data are provided as a Source Data file.

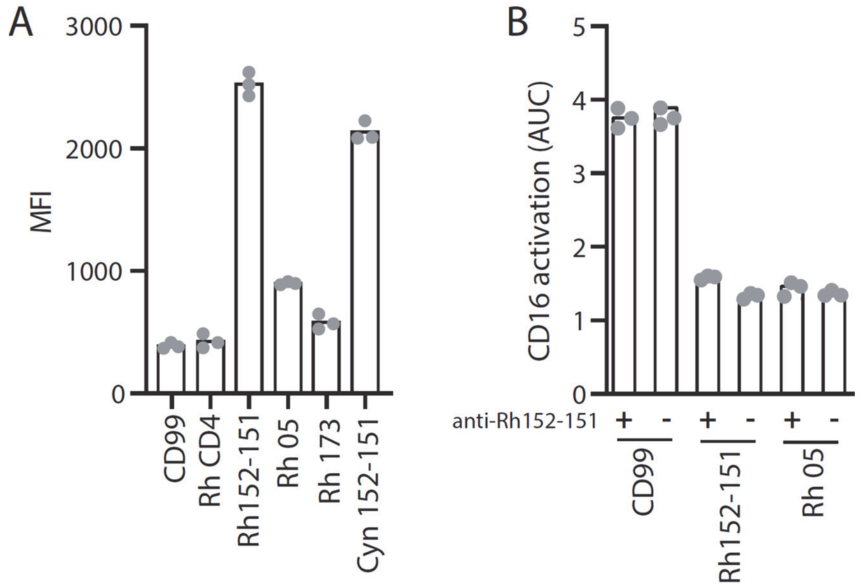

Fig S3. A Rh152/151 specific mAb from mouse immunization does not impact vFcγR function. A) HeLa cells transfected with RhCMV vFcγRs or control proteins (rhesus-CD4, human CD99) were probed for recognition by the PE-labeled Rh152-151 specific mAb via flow cytometry. Cyn = Cynomolgus. Rh = Rhesus. Symbols show mean fluorescence intensity (MFI) from independent experiments. Bar graphs show means from independent experiments.

B) Human CD16 activation was tested on HeLa cells transfected with rhesus-CD4 and the indicated vFcγRs or a human CD99 control from T2A-linked constructs to ensure equimolar expression. Cells were pre-incubated with the Rh152-151 specific mAb or not before addition of graded amounts of rhesus-CD4 specific mAb and co-culture with human CD16 reporter cells. Symbols show mean area under curve values (AUC) from independent experiments performed in technical replicates. Bar graph shows means from independent experiments. Source data are provided as a Source Data file.

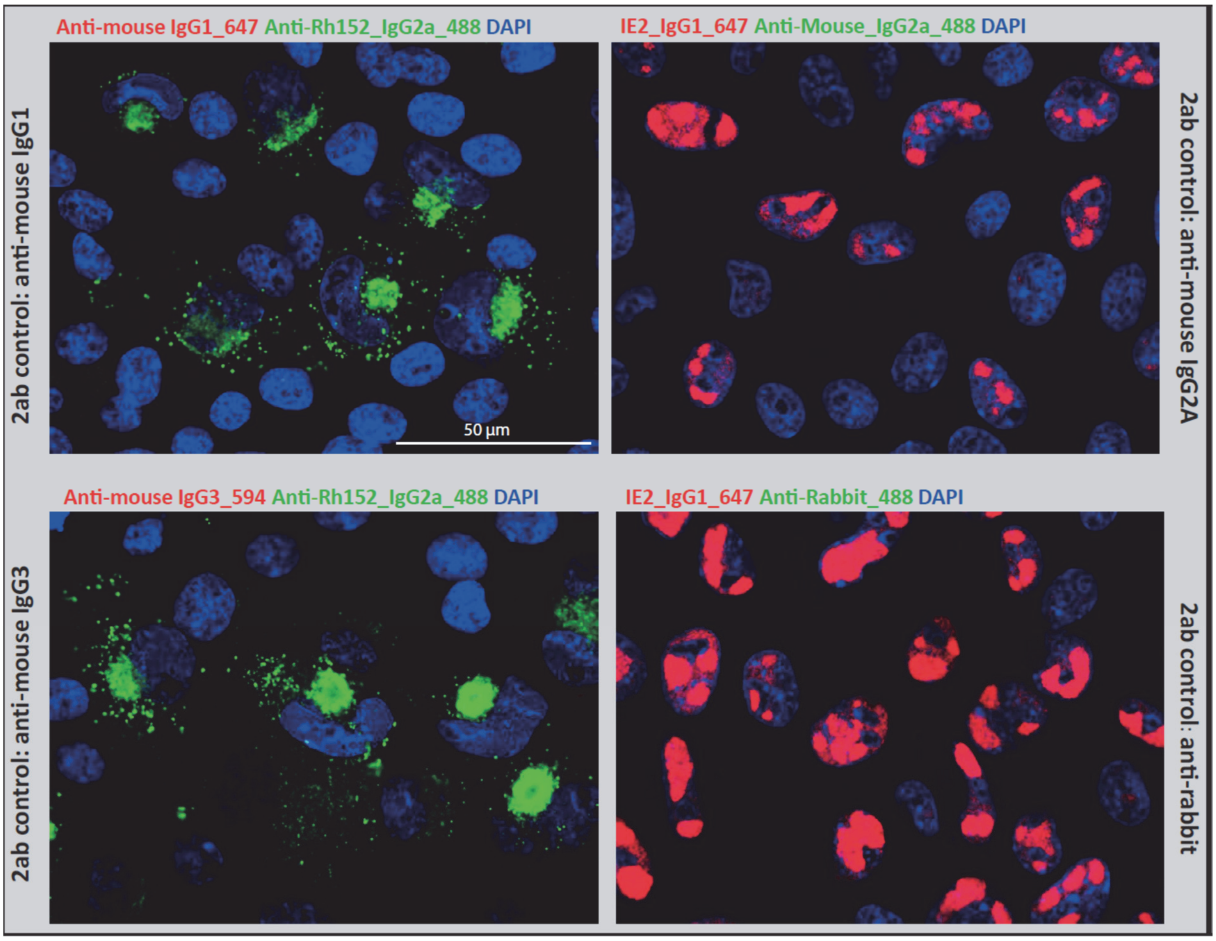

Fig S4. Control IFA for secondary antibody binding

To investigate whether secondary antibodies could bind to vFcγRs and result in non-specific signals, tRFs were infected with FL-RhCMV/Rh05-HA/Rh173-V5 at MOI 1 and IFA was performed at 48 hpi. Cells were probed using either anti-IE2 or anti-RhCMV antibodies for detecting infected cells, and additionally also probed with all florescent conjugated secondary antibodies. No non-specific vFcγR binding of secondary antibodies was observed in infected cells.

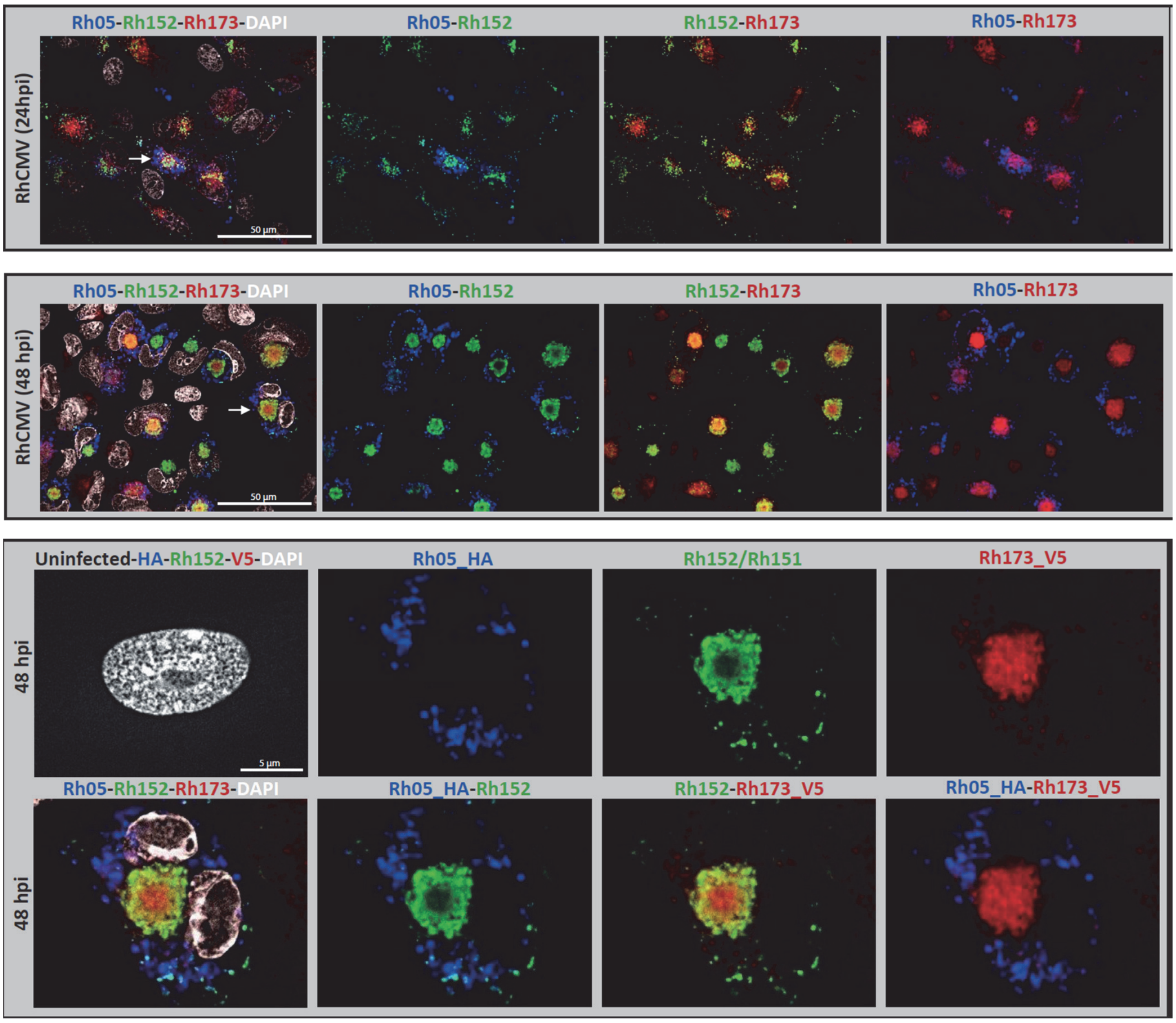

Fig S5. IFA for vFcγR co-localization at 24h post-infection

tRFs were infected with RhCMV FL+Rh05-HA+Rh173-V5 at MOI 1 and immunofluorescence assay was performed at 24 hpi and 48 hpi. Cells were probed using anti-HA, anti-Rh152/151 and anti-V5 antibodies for detecting vFcγRs. Data presented in the top row is a 100X microscopic view image at 24 hpi (cropped images in Fig. 3F), while the center row shows the 100X microscopic view image at 48 hpi. The bottom row shows a cropped representative single cell at 48 hpi (marked with white arrow).

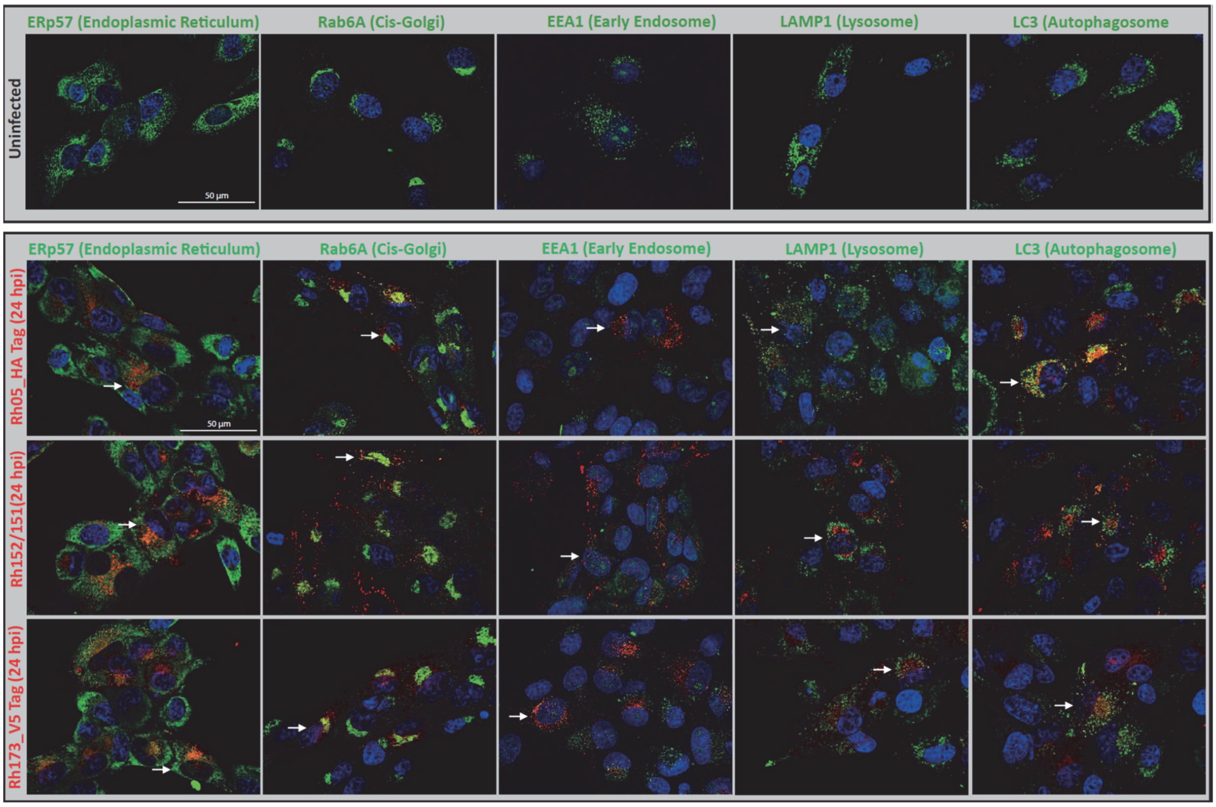

Fig S6. Co-localization of vFcγRs with markers of cellular organelles

Organelle staining pattern in uninfected tRF cells are shown in top row. In bottom row, tRFs were infected with FL-RhCMV/Rh05-HA/Rh173-V5 at MOI 1 and IFA was performed at 24 hpi. Cells were probed using anti-HA, anti-Rh152/151 and anti-V5 antibodies for detecting vFcRs. Additionally, cells were probed using anti-ERp57, Rab6A, LAMP1 and LC3 for detecting specific organelles. Data presented in bottom row is the complete 100X microscopic view image at 24 hpi (cropped images in Fig. 3G).

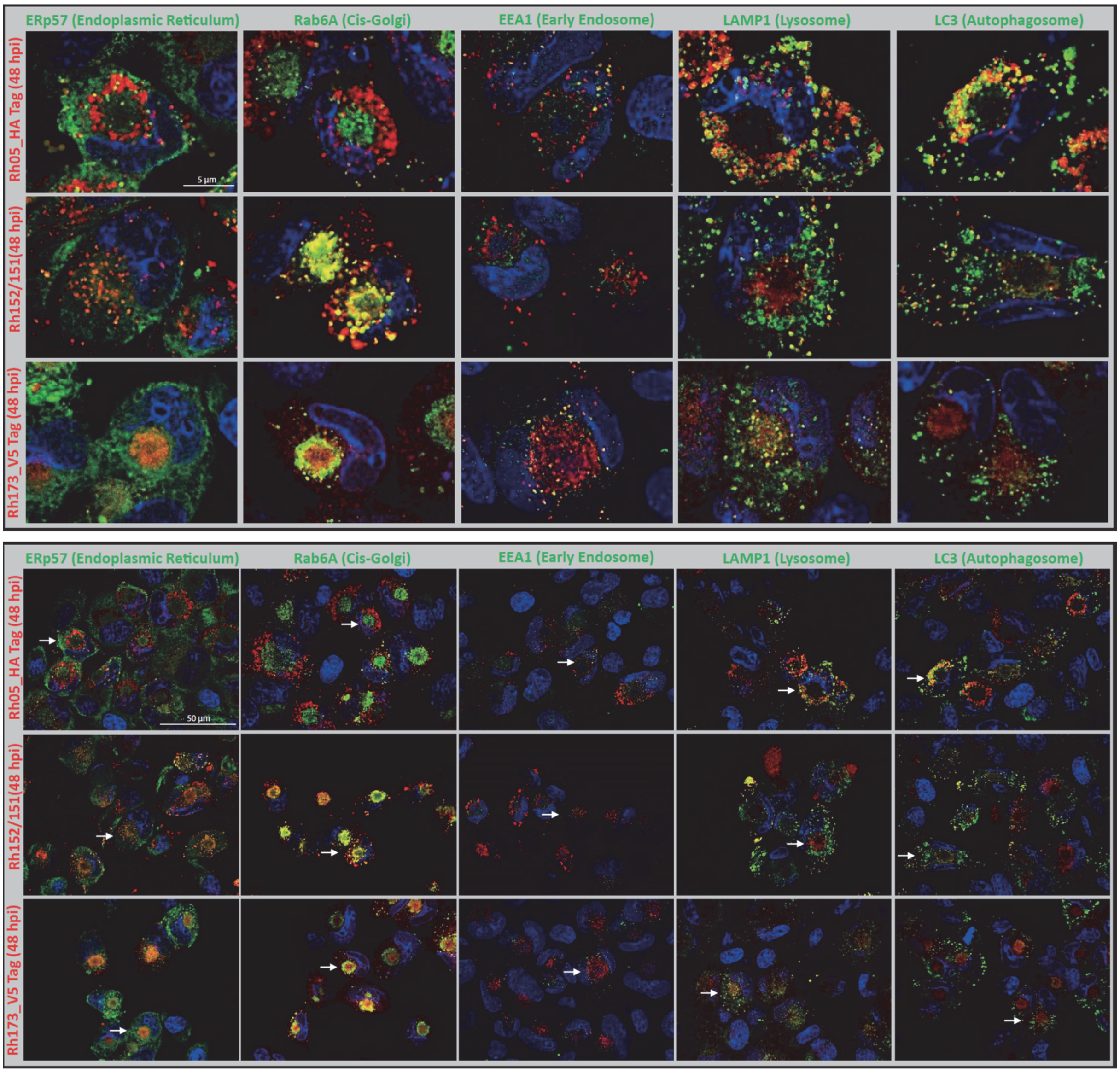

Fig S7. IFA for vFcγR co-localization at 24h post-infection

tRFs were infected with FL-RhCMV/Rh05-HA/Rh173-V5 at MOI 1 and immunofluorescence assay was performed at 48 hpi. Cells were probed using anti-HA, anti-Rh152/151 and anti-V5 antibodies for detecting vFcRs. Additionally, cells were probed using anti-ERp57, Rab6A, LAMP1 and LC3 for detecting specific organelles. The data presented in top panel is cropped representative single cell view from 100X microscopic view image. Bottom row shows cropped representative single cell at 48 hpi (marked with white arrow).

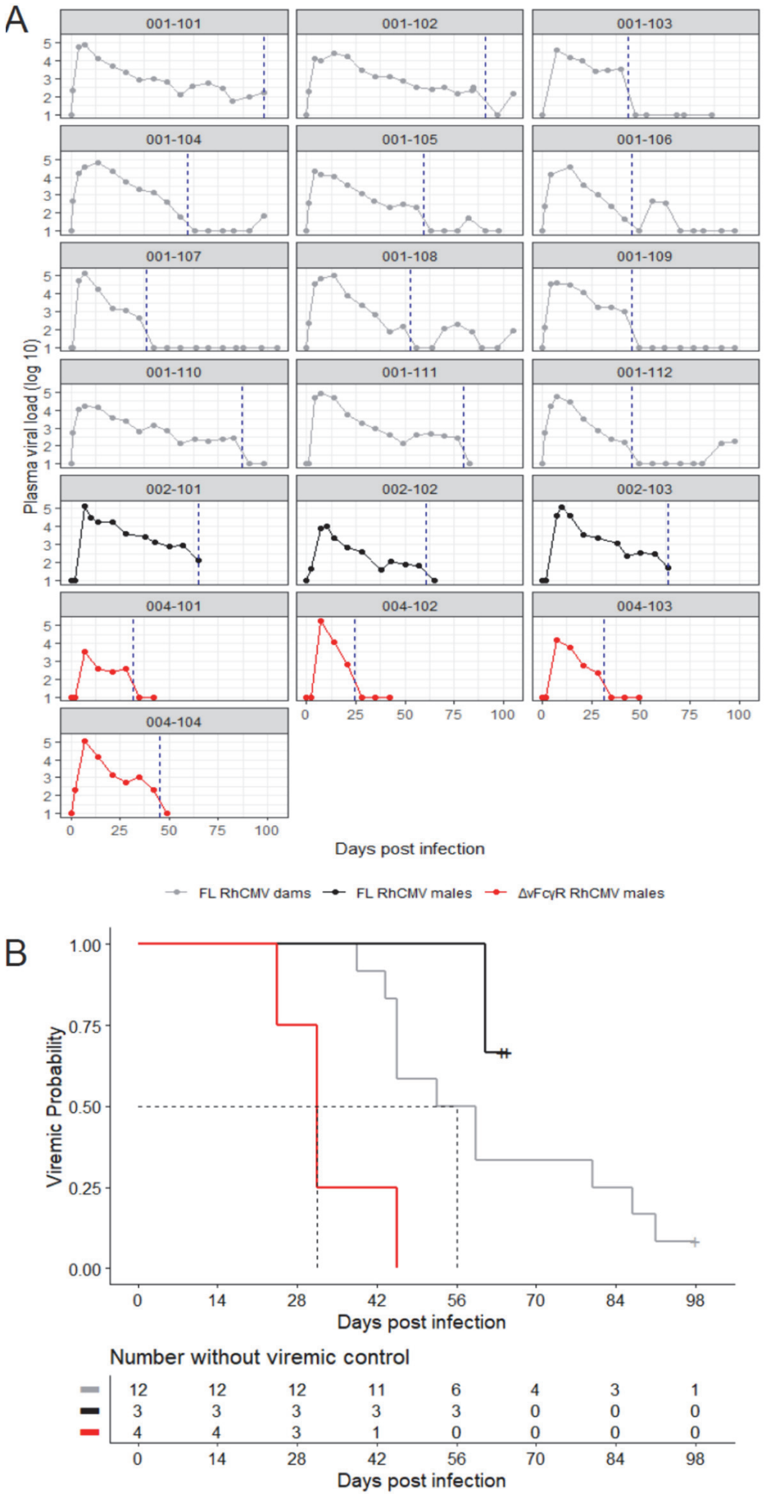

Fig S8. Sensitivity analyses of time to viral control

A) Viral genome copy number were determined by qPCR using primer/probe sets targeting exon 1 of the IE locus and reported via interpolation of a standard curve. The timing of control of DNAemia was estimated by the midpoint between the last day where VL was above the limit-of-detection and the first day observed after peak DNAemia where VL fell below the limit-of-detection. Values below the limit-of-detection were set to 1 for visualization. B) Kaplan-Meyer survival analysis comparing animals infected with FL-RhCMV (n = 3 males and n = 12 dams) versus FL-RhCMVΔΔΔ (n = 4 males). The median time to control was 31.5 days post infection in male animals infected with FL-RhCMVΔΔΔ compared to 56 days in dams infected with FL-RhCMV. Median time to control was not calculable in males infected with FL-RhCMV because only 1 of the 3 met the definition of control prior to the end of the sampling schedule for this group, maintaining statistical significance between the two groups of males despite the reduction in sample size ($p = 0.029$).

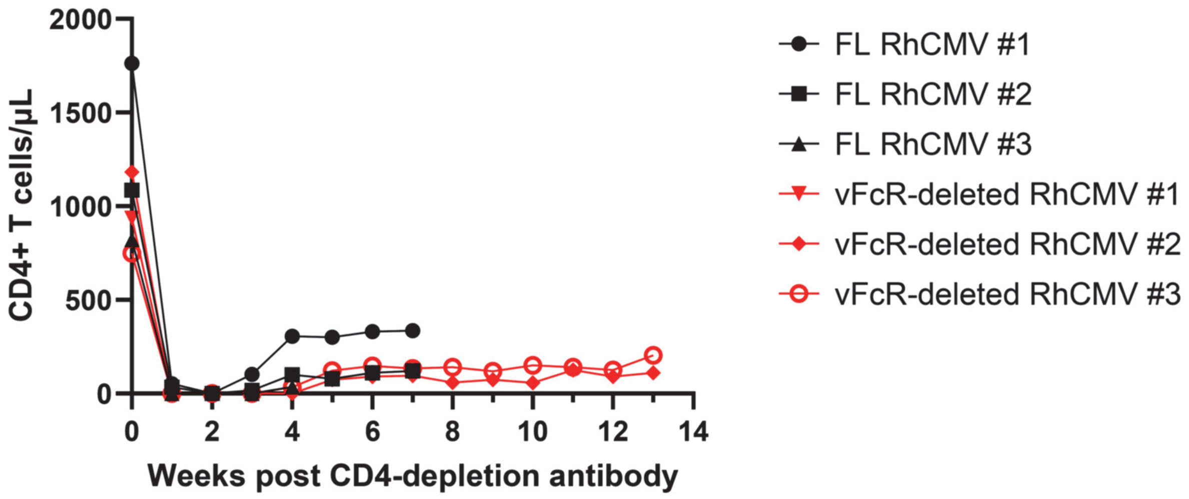

Fig S9. CD4-depletion in RM dams

Efficiency over time of CD4 antibody depletion in 6 RM dams used in Fig. 5E (Symbols are matched between Figures). We confirmed CD4+ T cell depletion via flow cytometry determination of cell counts normalized to the volume of sample blood. Depletion was sustained in all animals throughout the sampling period.

Source data are provided as a Source Data file.

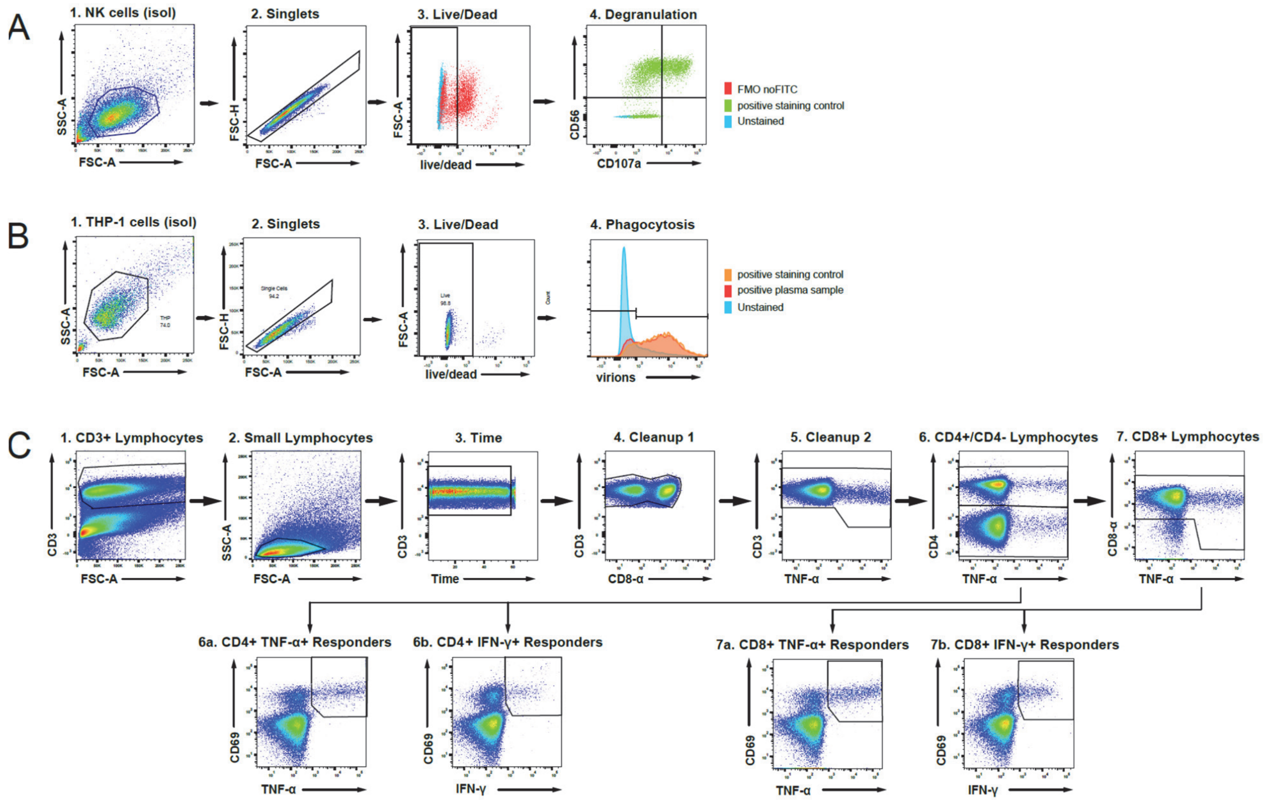

Fig. S10. Gating Strategies

Flow cytometry gating strategies for A) antibody-dependent cytolysis (ADCC), B) antibody-dependent phagocytosis (ADCP) and C) T cell activation assays.
