# Supplementary material for: Rhesus Cytomegalovirus-encoded Fcγ-binding glycoproteins facilitate viral evasion from IgG-mediated humoral immunity
Source: Nat Commun. 2025 Jan 31;16:1200. doi: 10.1038/s41467-025-56419-3 (PMC11782611; doi:10.1038/s41467-025-56419-3)
Supplement: Supplementary file 2 — Reporting Summary [file 41467_2025_56419_MOESM2_ESM.pdf]

nature portfolio

Corresponding author(s): Daniel Malouli
Philipp Kolb

Last updated by author(s): Oct 11, 2024

Reporting Summary

Nature Portfolio wishes to improve the reproducibility of the work that we publish. This form provides structure for consistency and transparency in reporting. For further information on Nature Portfolio policies, see our Editorial Policies and the Editorial Policy Checklist.

Statistics

For all statistical analyses, confirm that the following items are present in the figure legend, table legend, main text, or Methods section.

n/a	Confirmed	
☐	☒	The exact sample size (*n*) for each experimental group/condition, given as a discrete number and unit of measurement
☐	☒	A statement on whether measurements were taken from distinct samples or whether the same sample was measured repeatedly
☐	☒	The statistical test(s) used AND whether they are one- or two-sided<br>*Only common tests should be described solely by name; describe more complex techniques in the Methods section.*
☐	☒	A description of all covariates tested
☐	☒	A description of any assumptions or corrections, such as tests of normality and adjustment for multiple comparisons
☐	☒	A full description of the statistical parameters including central tendency (e.g. means) or other basic estimates (e.g. regression coefficient) AND variation (e.g. standard deviation) or associated estimates of uncertainty (e.g. confidence intervals)
☐	☒	For null hypothesis testing, the test statistic (e.g. *F*, *t*, *r*) with confidence intervals, effect sizes, degrees of freedom and *P* value noted<br>*Give P values as exact values whenever suitable.*
☒	☐	For Bayesian analysis, information on the choice of priors and Markov chain Monte Carlo settings
☒	☐	For hierarchical and complex designs, identification of the appropriate level for tests and full reporting of outcomes
☒	☐	Estimates of effect sizes (e.g. Cohen's *d*, Pearson's *r*), indicating how they were calculated

Our web collection on statistics for biologists contains articles on many of the points above.

Software and code

Policy information about availability of computer code

Data collection	iSeq 100 Software Tools used for collecting NGS data sets on the Illumina iSeq.<br>The software provided for the QuantStudio 7 Flex Real-Time PCR System was used to collect qPCR data.<br>The software provided with the Keyence BZ-X710 microscope was used to capture images of infected cells.<br>EVOS fluorescence microscope software was used to capture pictures of infected cells for FFU determination.<br>FlowJo Software was used to evaluate flow cytometry data
Data analysis	Geneious Prime® 2024.0.5 used for designing homologous recombination strategies to construct the vectors used in this study. We also used this software to quality control all vectors either by Sanger sequencing or by next generation sequencing.<br>The FIJI software was used processed images created using the Keyence BZ-X710 microscope.<br>GraphPad Prism was used to analyze qPCR results and the perform statical analyses.<br>The ImageJ software was used to process the images generated on the EVOS fluorescence microscope and to count infected cells to determine FFUs.<br>R was used for statistical analysis

For manuscripts utilizing custom algorithms or software that are central to the research but not yet described in published literature, software must be made available to editors and reviewers. We strongly encourage code deposition in a community repository (e.g. GitHub). See the Nature Portfolio guidelines for submitting code & software for further information.

Data

Policy information about availability of data

All manuscripts must include a data availability statement. This statement should provide the following information, where applicable:

- Accession codes, unique identifiers, or web links for publicly available datasets
- A description of any restrictions on data availability
- For clinical datasets or third party data, please ensure that the statement adheres to our policy

Data will be made available on request if not given in the source data file.

Research involving human participants, their data, or biological material

Policy information about studies with human participants or human data. See also policy information about sex, gender (identity/presentation), and sexual orientation and race, ethnicity and racism.

Reporting on sex and gender	*Use the terms sex (biological attribute) and gender (shaped by social and cultural circumstances) carefully in order to avoid confusing both terms. Indicate if findings apply to only one sex or gender; describe whether sex and gender were considered in study design; whether sex and/or gender was determined based on self-reporting or assigned and methods used. Provide in the source data disaggregated sex and gender data, where this information has been collected, and if consent has been obtained for sharing of individual-level data; provide overall numbers in this Reporting Summary. Please state if this information has not been collected. Report sex- and gender-based analyses where performed, justify reasons for lack of sex- and gender-based analysis.*
Reporting on race, ethnicity, or other socially relevant groupings	*Please specify the socially constructed or socially relevant categorization variable(s) used in your manuscript and explain why they were used. Please note that such variables should not be used as proxies for other socially constructed/relevant variables (for example, race or ethnicity should not be used as a proxy for socioeconomic status). Provide clear definitions of the relevant terms used, how they were provided (by the participants/respondents, the researchers, or third parties), and the method(s) used to classify people into the different categories (e.g. self-report, census or administrative data, social media data, etc.) Please provide details about how you controlled for confounding variables in your analyses.*
Population characteristics	*Describe the covariate-relevant population characteristics of the human research participants (e.g. age, genotypic information, past and current diagnosis and treatment categories). If you filled out the behavioural & social sciences study design questions and have nothing to add here, write "See above."*
Recruitment	*Describe how participants were recruited. Outline any potential self-selection bias or other biases that may be present and how these are likely to impact results.*
Ethics oversight	*Identify the organization(s) that approved the study protocol.*

Note that full information on the approval of the study protocol must also be provided in the manuscript.

Field-specific reporting

Please select the one below that is the best fit for your research. If you are not sure, read the appropriate sections before making your selection.

☒ Life sciences ☐ Behavioural & social sciences ☐ Ecological, evolutionary & environmental sciences

For a reference copy of the document with all sections, see nature.com/documents/nr-reporting-summary-flat.pdf

Life sciences study design

All studies must disclose on these points even when the disclosure is negative.

Sample size	Samples size was originally determined with the intent to use the fewest number of animals needed to achieve meaningful results. The here presented in vivo experiments in rhesus macaques generated statistically significant and coherent results and hence the sample sizes are appropriate for the scientific questions asked.
Data exclusions	No data was excluded
Replication	All experiments were performed in triplicates and all experimental results repeated across individual experiments or as indicated in the legends
Randomization	All our animals were of comparable age and were house under similar conditions. We demonstrated that biological sex did not influence the phenotype observed. All male animals included in this study were randomly assigned to their cohorts.
Blinding	While macaque infections were not blinded and the research staff was informed which animal received what virus, samples were shipped to a secondary location from preparation and data analysis and here the samples were blinded and the research staff did not know which animal

received which virus.

Reporting for specific materials, systems and methods

We require information from authors about some types of materials, experimental systems and methods used in many studies. Here, indicate whether each material, system or method listed is relevant to your study. If you are not sure if a list item applies to your research, read the appropriate section before selecting a response.

Materials & experimental systems

n/a	Involved in the study
☐	☒ Antibodies
☐	☒ Eukaryotic cell lines
☒	☐ Palaeontology and archaeology
☐	☒ Animals and other organisms
☒	☐ Clinical data
☒	☐ Dual use research of concern
☒	☐ Plants

Methods

n/a	Involved in the study
☒	☐ ChIP-seq
☐	☒ Flow cytometry
☒	☐ MRI-based neuroimaging

Antibodies

Antibodies used	all antibodies are given in the materials and methods section
Validation	all antibodies are commerically validated as described by the supplier

Eukaryotic cell lines

Policy information about cell lines and Sex and Gender in Research

Cell line source(s)	primary rhesus fibroblasts (RF) and telomerized rhesus fibroblasts (tRF) derived from embryonic tissue of a rhesus macaque fetus. Gender not determined. (source: Oregon NPRC)<br>HEK293T cells (ATCC-CRL3216)<br>293T-CD20 (kind gift by Irving Chen, details in methods section)<br>Hela cells (ATCC-CCL2)<br>BW5147 mouse thymoma cells (origin descibed in methods section)<br>Rhesus retinal pigment epithelial (RPE) cells (gift from Tom Shenk, details in methods section)
Authentication	commercial or in previous publications (references in the methods section)
Mycoplasma contamination	All cells tested negative for mycoplasma contamination.
Commonly misidentified lines<br>(See ICLAC register)	None of the cells used in this study are commonly misidentified.

Animals and other research organisms

Policy information about studies involving animals; ARRIVE guidelines recommended for reporting animal research, and Sex and Gender in Research

Laboratory animals	The study involved the use of purpose bred, Indian-origin rhesus macaques of reproductive age.
Wild animals	Study did not involve wild animals.
Reporting on sex	Our work investigated the impact on the deletion of all identified viral Fcgamma receptors encoded by RhCMV on virus replication in CMV naive animals. We did not observe that the biological sex had an effect and the development of viremia in CMV naive animals and we thus used female animals from a pregnant cohort as control animals side by side but not merged with male control animals.
Field-collected samples	This study did not contain samples collected from the field.
Ethics oversight	All studies received full approval by the appropriate local IACUC and IBC committees.

Note that full information on the approval of the study protocol must also be provided in the manuscript.

Plants

Seed stocks	*Report on the source of all seed stocks or other plant material used. If applicable, state the seed stock centre and catalogue number. If plant specimens were collected from the field, describe the collection location, date and sampling procedures.*
Novel plant genotypes	*Describe the methods by which all novel plant genotypes were produced. This includes those generated by transgenic approaches, gene editing, chemical/radiation-based mutagenesis and hybridization. For transgenic lines, describe the transformation method, the number of independent lines analyzed and the generation upon which experiments were performed. For gene-edited lines, describe the editor used, the endogenous sequence targeted for editing, the targeting guide RNA sequence (if applicable) and how the editor was applied.*
Authentication	*Describe any authentication procedures for each seed stock used or novel genotype generated. Describe any experiments used to assess the effect of a mutation and, where applicable, how potential secondary effects (e.g. second site T-DNA insertions, mosiacism, off-target gene editing) were examined.*

Flow Cytometry

Plots

Confirm that:

- [x] The axis labels state the marker and fluorochrome used (e.g. CD4-FITC).
- [x] The axis scales are clearly visible. Include numbers along axes only for bottom left plot of group (a 'group' is an analysis of identical markers).
- [ ] All plots are contour plots with outliers or pseudocolor plots.
- [ ] A numerical value for number of cells or percentage (with statistics) is provided.

Methodology

Sample preparation	cell lines harvested from culture and stained in PBS/3%FCS
Instrument	LSR Fortessa BD
Software	FACS Diva. FlowJo
Cell population abundance	derived from transfected cell lines. Highest purity
Gating strategy	FSC-A SSC-A // FSC-A FSC-H (duoblets) // live cells (DAPI) // GFP (transfected cells) // Histograms of target fluorophore normalized to mode. Evaluation of MFI.

- [ ] Tick this box to confirm that a figure exemplifying the gating strategy is provided in the Supplementary Information.
